# Acute Stanford Type A Aortic Dissection After Transcatheter Aortic Valve Implantation Necessitating the Bentall Procedure: A Case Report

**DOI:** 10.7759/cureus.107957

**Published:** 2026-04-29

**Authors:** Nobu Yokoyama, Kosuke Miyoshi, Daigo Shinoda, Manabu Shiraishi

**Affiliations:** 1 Department of Cardiovascular Surgery, Tokyo Metropolitan Bokutoh Hospital, Tokyo, JPN

**Keywords:** aortic stenosis (as), bentall procedure, pop-out, stanford type a acute aortic dissection, transcatheter aortic valve implantation (tavi)

## Abstract

An 80-year-old female with a history of sarcoidosis, dyslipidemia, hypertension, and permanent pacemaker implantation for complete atrioventricular block, presented with severe calcified aortic stenosis, corresponding to New York Heart Association Class II-III heart failure. The patient subsequently underwent elective transcatheter aortic valve implantation (TAVI) using a self-expandable transcatheter heart valve. After valve deployment, transesophageal echocardiography revealed a flap in the ascending aorta. Contrast-enhanced computed tomography (CECT) confirmed the diagnosis of Stanford Type A acute aortic dissection, and emergency surgery was indicated based on the presumed injury to the sinus of Valsalva caused by pop-out and recapture maneuvers during TAVI. Intra-operative findings revealed a transverse intimal tear extending across the right coronary and non-coronary cusps, necessitating aortic root replacement (Bentall procedure) and concomitant coronary artery bypass grafting to the right coronary artery.

## Introduction

Transcatheter aortic valve implantation (TAVI) has evolved as a minimally invasive treatment for severe aortic stenosis, involving the delivery and deployment of a transcatheter heart valve across the native aortic valve under fluoroscopic and echocardiographic guidance. It is now increasingly considered a first-line treatment for severe aortic stenosis [1]. The PARTNER 2 trial (Placement of AoRTic TraNscathetER Valves 2), a landmark multicenter randomized study, demonstrated that TAVI is non-inferior to surgical aortic valve replacement (SAVR) in patients with severe aortic stenosis at intermediate surgical risk, with respect to the endpoint of death from any cause or disabling stroke, even at 5-year follow-up [2]. Even in clinical trials involving low-risk patients, TAVI has been shown to be non-inferior to SAVR in terms of 30-day mortality (0.5% vs. 1.3%) and even superior with respect to outcomes such as disabling stroke and bleeding complications [3]. Based on these findings, TAVI has been established as a standard therapeutic option, and its indications have continued to expand. As the technique has evolved, the incidence of TAVI-related complications that require conversion to emergency open-heart surgery has decreased [3]. However, once conversion becomes necessary, the procedure has a high risk of mortality, with a reported rate as high as 22.3% [4].

Acute aortic dissection is one of the most serious complications associated with TAVI, and it may require conversion to surgical intervention. In particular, Stanford type A aortic dissection associated with TAVI is considered to be caused by direct mechanical injury to the aortic wall around the aortic root from the implanted device or balloon manipulation before or after deployment, and is often associated with worse outcomes than spontaneous acute aortic dissection [5]. Nevertheless, due to its low incidence and differences in etiology from typical acute aortic dissections, no consensus has been established regarding optimal treatment strategies [5-7]. Therefore, prompt and appropriate management tailored to the extent of the dissection and its underlying mechanism is essential on a case-by-case basis. We report a case of acute aortic dissection extending to the aortic root after TAVI, likely caused by sinus of Valsalva injury involving both the right coronary and non-coronary cusps during device deployment.

## Case presentation

The patient was an 80-year-old female with a medical history of sarcoidosis, complete atrioventricular block that required dual-chamber permanent pacemaker implantation, hypertension, and dyslipidemia. She presented with worsening exertional dyspnea and dizziness. Upon evaluation, she was diagnosed with severe aortic stenosis, complicated by heart failure, corresponding to New York Heart Association functional Class II-III [8]. Transthoracic echocardiography revealed a heavily calcified aortic valve with a peak velocity of 4.1 m/s, a mean transvalvular gradient of 37 mmHg, a stroke volume index of 47 mL/m², and an aortic valve area of 0.51 cm². Left ventricular systolic function was preserved, with an ejection fraction of 60%. Coronary computed tomography (CT) revealed no significant stenosis, and vascular access was favorable.

**Figure 1:**
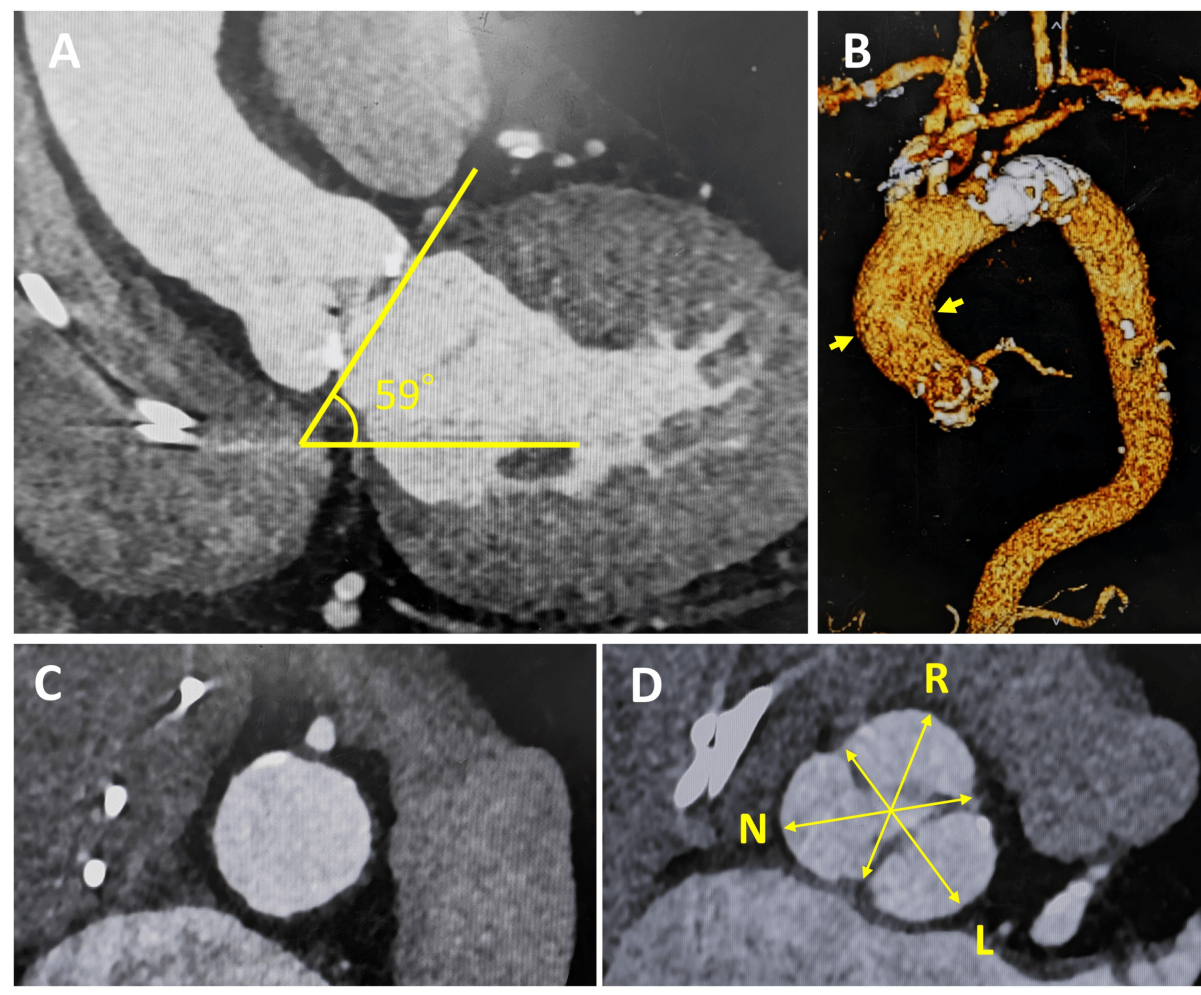
Findings on preoperative computed tomography (A) The aortic root demonstrated an angulation of 59°. (B) A sharp curvature extending into the proximal ascending aorta (yellow arrow) . (C) Minimal calcification at the sinotubular junction. (D) The aortic valve was tricuspid, and the sinus of Valsalva diameters were 25.6 mm (R: right coronary cusp), 27.9 mm (L: left coronary cusp), and 27.9 mm (N: non-coronary cusp).

However, the aortic root demonstrated a relatively steep angulation of 59°, with a sharp curvature extending into the proximal ascending aorta, without significant tortuosity. The sinotubular junction showed only minimal calcification. The sinus of Valsalva diameters were 25.6 mm (right coronary cusp), 27.9 mm (left coronary cusp), and 27.9 mm (non-coronary cusp) (Figure 1). Given the patient’s advanced age, sarcoidosis, and other comorbidities, the Society of Thoracic Surgeons (STS) risk score was 4.0%, indicating an elevated surgical risk. Therefore, we performed a multidisciplinary Heart Team evaluation. In accordance with the clinical practice guidelines of the American College of Cardiology (ACC) and the American Heart Association (AHA) [9], which provide evidence-based recommendations for the diagnosis, management, and prevention of cardiovascular diseases, TAVI was selected in preference over SAVR.

Elective TAVI was performed using a self-expandable transcatheter heart valve (THV) (Evolut FX; Medtronic, Minneapolis, MN, USA). Following balloon aortic valvuloplasty, the THV was deployed under controlled pacing. During deployment at approximately 60%, the device exhibited a pop-out phenomenon due to aortic flow, and it was promptly recaptured. The pop-out phenomenon was considered to be multifactorial. Relatively slow controlled pacing at approximately 120 bpm to avoid hypotension may have increased the influence of antegrade blood flow, and the initial deployment position may have been slightly higher than intended. In addition, steep aortic root angulation likely contributed to the displacement of the transcatheter heart valve toward the greater curvature due to the stiffness of the guidewire. Consequently, during pop-out, the proximal edge of the stent sprang back towards the aortic side, and it came into contact with the sinus of Valsalva.

**Figure 2:**
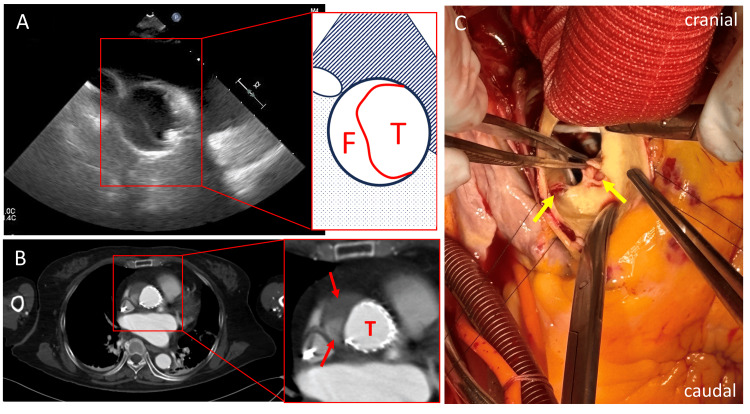
Aortic dissection and intimal tears (A) Transesophageal echocardiography and (B) Contrast-enhanced computed tomography revealing Stanford type A acute aortic dissection. T: true lumen; F: false lumen; red curve indicates mobile intimal flap; upper red arrow indicates a hematoma within the false lumen; lower red arrow indicates contrast enhancement within the false lumen. (C) Intimal tears are in the sinus of the right coronary cusp (right arrow) and non-coronary cusp (left arrow).

To prevent further pop-out, the THV was deliberately deployed at a deeper position, and the procedure was completed successfully. However, post-procedural transesophageal echocardiography revealed a mobile intimal flap in the ascending aorta distal to the implanted TAVI device (Figure 2A). Evaluation of the aortic root at the level of the device was limited by acoustic shadowing from the valve frame, and the proximal extent of the dissection could not be clearly determined. As there was no pericardial effusion and hemodynamics remained stable, contrast-enhanced CT was performed, which confirmed Stanford Type A acute aortic dissection extending from the aortic root to the arch of the aorta (Figure 2B). Contrast-enhanced computed tomography did not identify a definite entry tear. The dissection flap extended at least to the level of the non-coronary cusp, with contrast enhancement within the false lumen at this level.

The maximum false lumen thickness was 17 mm, with additional enhancement in the proximal ascending aorta. Coronary opacification was preserved, with no evidence of coronary obstruction. If the entry tear had been located in the ascending aorta due to the distal edge of the device, conservative management or surgical treatment with ascending aortic replacement and aortic valve replacement might have been considered. However, in this case, the dissection extended proximally into the sinus of Valsalva, suggesting that the entry tear was located at this level and carried a high risk of coronary artery obstruction and cardiac tamponade. After a multidisciplinary heart team discussion, and given that the mortality risk associated with conservative management was considered to be greater than the surgical risk, emergency surgery was performed with a plan for aortic root replacement.

**Figure 3:**
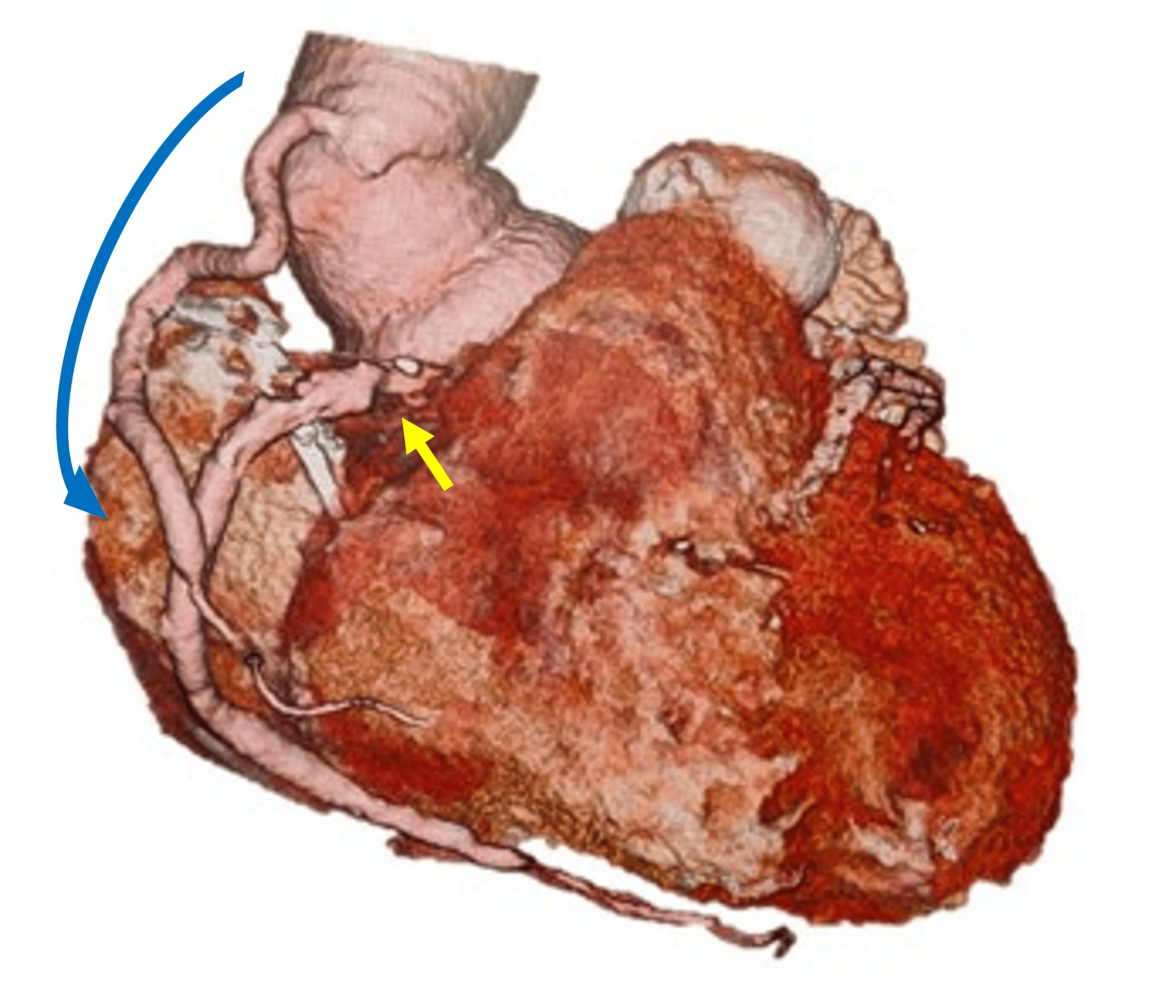
Coronary computed tomographic angiography after surgery Coronary artery bypass grafting from the vascular graft to the right coronary artery was performed (blue arrow). The proximal portion of the right coronary artery was ligated (yellow arrow).

The surgery was performed via median sternotomy under moderate hypothermic circulatory arrest. To avoid interference with the proximal anastomosis and to evaluate the presence of a proximal intimal tear, the THV was removed. For removal, the distal end of the THV was grasped at multiple points with forceps, it was compressed, and carefully twisted out, allowing for relatively smooth extraction. Subsequent inspection of the aortic lumen revealed two transverse intimal tears, each approximately 15 mm in length, in the sinus of Valsalva beneath the right coronary orifice, and in the sinus of the non-coronary cusp (Figure 2C). As resection of the tear required replacement of the aortic root, a modified Bentall procedure using a bioprosthetic valve was performed [10]. The left coronary orifice was reconstructed using the Carrel patch technique; however, the right coronary orifice could not be anatomically reconstructed because of an extension of the dissection from the tear. Consequently, the proximal portion of the right coronary artery was ligated, and coronary artery bypass grafting to the right coronary artery was performed using a great saphenous vein graft (Figure 3).

The postoperative course was uneventful, and the patient recovered steadily. The patient was extubated on postoperative day 3 and was discharged in good condition on postoperative day 16.

## Discussion

Transcatheter aortic valve implantation is increasingly used as an effective alternative to SAVR in patients with severe aortic stenosis, including those with a low surgical risk [11]. Despite a decline in complications that require emergency open-heart surgery as a result of broader indications and technical improvements [12], the presence of these complications remains potentially fatal. According to Verolino et al., the incidence of emergency open-heart surgery due to intra-procedural complications during TAVI is approximately 1.3% (224 of 17,473 patients), with a high mortality rate of 22.3% (50 of 224 patients) following emergency surgery [4].

Acute aortic dissection, particularly Stanford Type A, is a rare but catastrophic complication of TAVI, with reported incidence rates typically ranging between 0.1% and 1.9% in major registries. Although infrequent, it is associated with high mortality and often necessitates emergent surgical intervention [7]. Ashwat et al. reported that although the incidence of dissection after TAVI was relatively low (nine of 4,317 patients, 0.2%), five of six patients who developed Type A dissection died [5]. Similarly, Hein et al. reported a mortality rate of 80% (four of five patients) for patients with Type A aortic dissection following TAVI who underwent surgical treatment [13]. Conversely, Hiruma et al. reported that conservative management was selected for six patients with a Type A dissection among 13 patients with a post-TAVI dissection because of advanced age and high surgical risk, and there were no aortic-related deaths [6].

Previous reports have suggested that, in balloon-expandable valves, oversizing of the delivery balloon in patients with a narrow and calcified sinotubular junction may be a risk factor for dissection [14]. In addition, for self-expandable valves, pre- and post-balloon dilation have also been proposed as potential risk factors. However, no clear difference in the incidence of dissection between self-expandable and balloon-expandable valves has been demonstrated [15]. In general, emergency surgery is the standard treatment for Stanford Type A acute aortic dissection [8]; however, due to its low incidence and the high mortality associated with surgery, there is no clear consensus regarding the optimal treatment strategy for Type A dissection following TAVI.

In the present case, contrast-enhanced CT revealed a dissection originating at the aortic root, without thrombus formation in the false lumen, prompting emergency surgery. Intra-operatively, there was no intimal tear in the ascending aorta; however, there was injury to the sinus of Valsalva, accompanied by two transverse intimal tears. Conservative treatment would most likely have resulted in life-threatening complications, such as cardiac tamponade. The tears were along the right coronary and non-coronary cusps, which is consistent with mechanical injury caused by the proximal edge of the self-expandable THV being pressed against the greater curvature of the Valsalva sinus during pop-out. In self-expandable valves, a steep aortic angulation has been suggested as a potential risk factor for delayed aortic dissection due to contact between the distal edge of the device and the aortic wall [14].

However, in the present case, the entry tear was located at the sinus of Valsalva, suggesting a different underlying mechanism. Although within the sizing indications, the relatively narrow Valsalva sinus diameter and steep aortic root angulation of 59 degrees may have contributed to pop-out and the risk of sinus injury. While reports of Stanford Type A dissection caused by pop-out of self-expandable THVs are limited [5], no previous study has explicitly described injury to the sinus of Valsalva.

Pop-out of a self-expandable THV during deployment accounts for approximately 20.5% of repositioning cases [16]. While there was no significant difference in mortality between cases with and without repositioning, in that report, the deep implantation depth (ID) was identified as a risk factor for permanent pacemaker implantation when a self-expandable THV was used [17]. Consequently, a high ID is often used to mitigate conduction disturbances. Nevertheless, despite its rarity, clinicians should remain vigilant for potentially fatal complications associated with pop-out. Rapid pacing has been reported to assist in achieving a high ID even with a self-expandable THV [18]; therefore, it may represent a useful technique for minimizing the risk of pop-out. However, a relatively high ID has also been reported to increase the risk of coronary obstruction and paravalvular leakage.

Recently, achieving an appropriate ID, taking into account commissural and/or coronary alignment, has been increasingly recognized as important [19]. Accordingly, the optimal ID should be determined based on individual patient characteristics, including anatomical features and underlying conditions. In cases such as the present one, involving patients with a pre-existing pacemaker, the potential benefit of a high ID may be limited. Therefore, in this case, planning a relatively deeper ID strategy from the outset might have been preferable to reduce complications associated with pop-out.

## Conclusions

In this case, a type A acute aortic dissection occurred during TAVI, with intimal tears involving both the right and non-coronary cusps of the sinus of Valsalva. The tears were oriented transversely, consistent with the direction of the proximal edge of a self-expandable THV. These findings suggest that the dissection may have been caused by mechanical interaction between the aortic wall and the proximal edge of the self-expandable THV during deployment. Prompt recognition and emergency surgical intervention, including a modified Bentall procedure, resulted in a favorable outcome. TAVI offers excellent therapeutic value due to its minimally invasive nature, particularly in older patients with significant comorbidities who are considered high-risk surgical candidates. However, these patients often have limited physiological reserve, and complications may result in fatal outcomes.

Type A aortic dissection after TAVI remains a rare but potentially catastrophic complication, and an optimal treatment strategy has not been clearly established. Therefore, management should be individualized based on the extent of dissection, the suspected location of the intimal tear, and imaging findings such as contrast enhancement in the false lumen. Although surgical repair carries inherent risks, emergency surgical intervention may be warranted when critical structures, such as the sinus of Valsalva, are involved.
